# Prediction of the targets of the main components in blood after oral administration of *Xanthii Fructus*: a network pharmacology study†

**DOI:** 10.1039/c8ra00186c

**Published:** 2018-02-27

**Authors:** Yanshuang Zhuang, Kunming Qin, Bing Yang, Xiao Liu, Baochang Cai, Hao Cai

**Affiliations:** Engineering Center of State Ministry of Education for Chinese Medicine Processing, Nanjing University of Chinese Medicine Nanjing 210023 China haocai_98@126.com 295331981@qq.com bingbingyang_2012@163.com 1498223254@qq.com +86-25-68193500 +86 13770500190 +86 13585148874; Nanjing Haichang Chinese Medicine Group Co., Ltd. Nanjing 210061 China qinkm123@126.com baochangcai_2012@126.com; Nanjing Haiyuan Prepared Slices of Chinese Crude Drugs Co., Ltd. Nanjing 210061 China; Huaihai Institute of Technology Lianyu Gang 222005 China

## Abstract

*Xanthii Fructus* (XF), a famous traditional Chinese medicine (TCM), has been widely used in the treatment of rhinitis and other diseases. However, the targets of the main XF components found in the blood after oral administration of XF extract are still unclear. In the current study, a feasible systems pharmacology method was developed to predict these targets. In accordance with our previous research, XF components were selected including cleomiscosin A, myristic acid, succinic acid, xanthosine, sitostenone, emodin, apigenin, and chrysophanol. Three components, namely emodin, apigenin, and chrysophanol, failed to be detected with target proteins, thus the other five components, namely cleomiscosin A, myristic acid, succinic acid, xanthosine and sitostenone, were eventually chosen for further systematic analysis. Ninety-nine target proteins and fifty-two pathways were found after a series of analyses. The frequency of some target proteins was much higher than that of others; high frequencies were obtained for P15086, P07360, P07195, MAOM_HUMAN (P23368), P35558, P35520, ACE_HUMAN (P12821), C1S_HUMAN (P09871), PH4H_HUMAN (P00439), FPPS_HUMAN (P14324), P50613, P12724, IMPA1_HUMAN (P29218), HXK1_HUMAN (P19367), P14061, and MCR_HUMAN (P08235). The frequency of eight pathways was also high, including Generic Transcription Pathway, RNA Polymerase II Transcription, Metabolism, Metabolism of steroids, Gene expression (Transcription), Cellular responses to stress, Platelet activation, signaling and aggregation, Signaling by Receptor Tyrosine Kinases, and Cellular Senescence. This study identified a common pathway – the Metabolism pathway – for all five XF components. We successfully developed a network pharmacology method to predict the potential targets of the main XF components absorbed in serum after oral administration of XF extract.

## Abstract 2

This paper developed a network pharmacology method to predict the potential pathways targeted by oral administration of XF extract.

## Graphical abstract

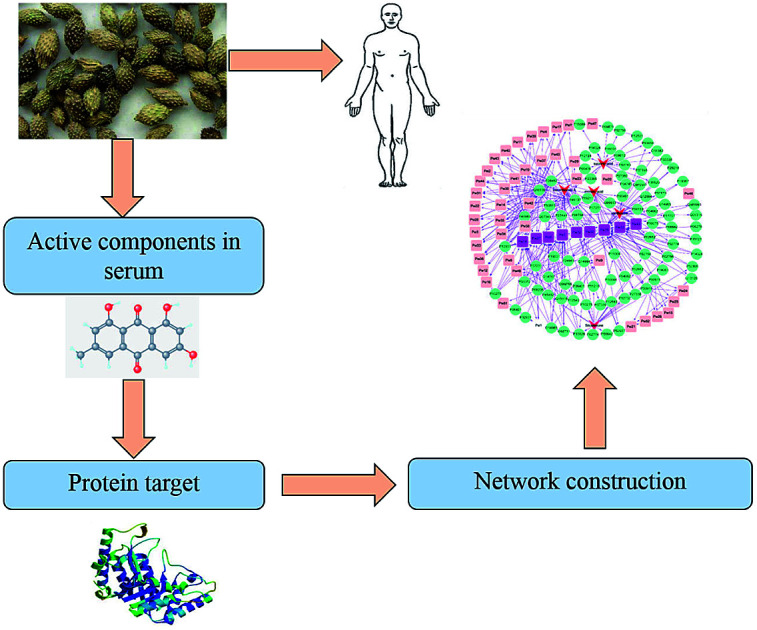

## 1. Introduction

Over thousands of years, abundant clinical experience has accumulated in the use of traditional Chinese medicine (TCM). TCM has exerted synergistic effects in the treatment of complex diseases with its multi-component properties and multi-target functioning, creating a difficult challenge for its modernization. Recently, network pharmacology has risen rapidly in the research field. It explores drug targets by finding the overall correlation between drugs and diseases when combined with systems biology, multidirectional pharmacology and multidisciplinary technology, such as in network analysis, computational biology and disease-gene–drug network construction. It could therefore provide a new approach for overcoming barricades in the way of TCM modernization.

Network pharmacology, based on the network of “disease-gene-target-drug” interactions, is a way of revealing the synergistic effects of complex drugs on the human system and finding efficient and low toxicity multi-target new drugs at the network level by observing the intervention of drugs and their impact on disease. With information databases such as gene network libraries, protein network libraries, disease network libraries, and drug network libraries, and systematic spectrogram data analysis, network pharmacology is able to reveal mysterious disease–disease, disease phenotype-target protein, target protein–drug and drug–drug linkages.^1–7^

Uncovering the material basis of TCM is the key and precondition for TCM quality control, which puts it at the core of TCM modernization. In a network pharmacology study, drug–drug networks can be constructed based on the similarities in the structures and efficacies of different drugs. In the process of TCM modernization, some researchers have achieved good initial results in exploring the essential properties of TCMs and revealing their comprehensive overall effects on multi-pathways, multi-targets and multi-components *via* the research ideas of network pharmacology.^8–11^

*Xanthii Fructus* (XF) is the ripe fruit of *Xanthium sibiricum Patr*. XF is used for the treatment of cramping, numbness of the limbs, ulcers, sinusitis, catarrhs, and pruritus, for its function in smoothing nasal orifices and eliminating wind-dampness.^12^ In modern clinic application, XF is commonly used for the treatment of rhinitis. Particularly when combined with *Magnoliae flos*, mint and other Chinese medicines, XF has enhanced effects in curing chronic rhinitis, allergic rhinitis and other rhinitis.^13^

## 2. Materials and methods

### 2.1 Screening active ingredients

**Fig. 1:**
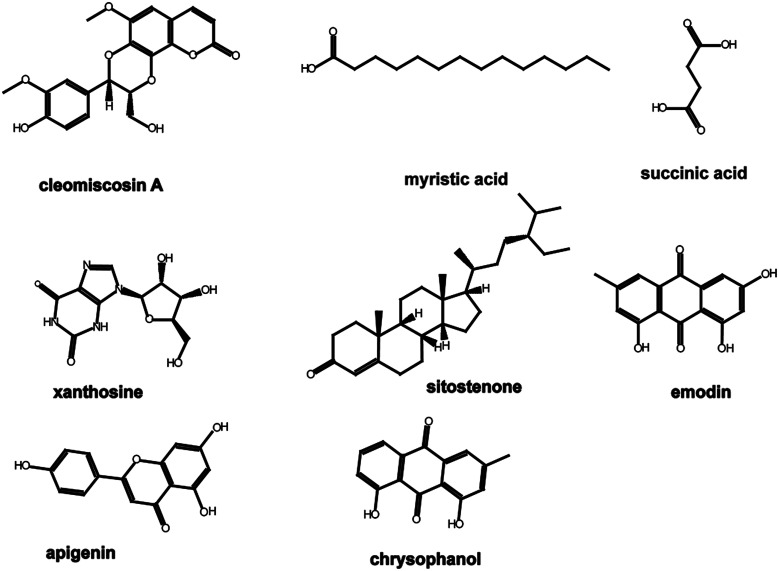
Structures of the components.

In our previous study (unpublished), components such as myristic acid, succinic acid, xanthosine, emodin, apigenin, and chrysophanol were identified from serum samples after oral administration of XF extracts. Components such as cleomiscosin A and sitostenone were filtered using the traditional Chinese medicine systems pharmacology (TcmSP™) database, and the parameters were set as follows: oral bioavailability (OB) ≥ 30%, drug-likeness (DL) ≥ 0.18. The structures of the components mentioned above are shown in Fig. 1.

### 2.2 Prediction of active component targets

Firstly, the MDL SD (*.sdf) type files of the above active ingredients were searched using the PubMed database. Secondly, targets, including information like the target name, matching value, target protein abbreviation, function, disease and applicable results related to the modified compound, could be predicted by importing each component file in *sdf format into the PharmMapper database. The top 20 high-matching targets, by value, were used as the TCM target proteins related to the components. The targets were then searched for in the UniProt database to identify human-related target codes.

### 2.3 Pathway comments and analysis

The retrieved target protein information was analyzed using the Reactome database to obtain the result of the related pathway “pathwayIdexByPathway_kegg”. A pathway was selected as reliable when its *P* value was less than 0.01.

### 2.4 Drug-target-pathway relationship

**Fig. 2:**
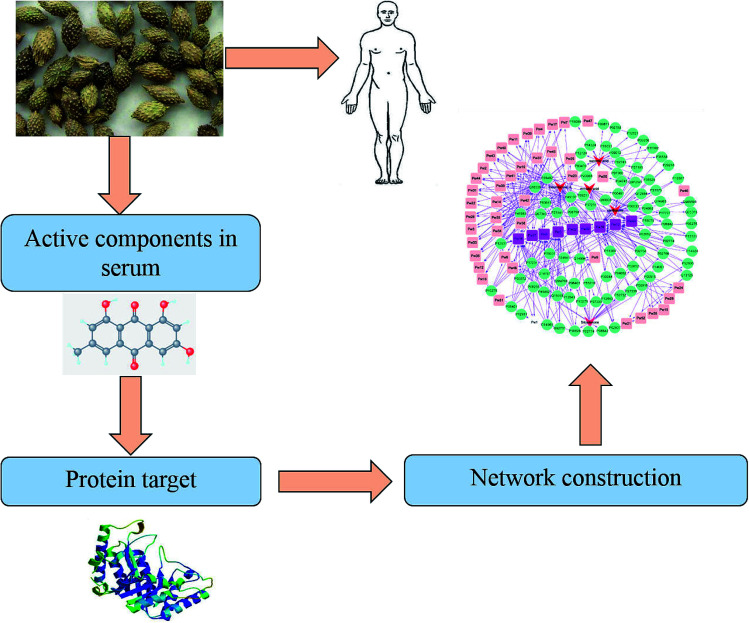
The whole framework of system pharmacology.

The predicted targets of five chemical components of XF, namely cleomiscosin A, myristic acid, succinic acid, xanthosine and sitostenone, were recorded in excel tables titled as ‘component-protein’ and ‘protein-pathway’. The tables were imported into Cytoscape software to construct the main effect components of the XF-target-pathway network. The network was mainly composed of three types of nodes: effect component, protein and pathway. The effect components and their related target proteins, and the proteins and their related pathways were all side-linked. When the target protein of the effect component was the same as the target protein of the pathway, the effect component was side-linked to the pathway. A complete network diagram was built by the establishment of connections including effect component-protein-pathway, effect component-protein-effect component, pathway-protein-pathway, protein-effect component-protein and other four kinds of connection. The whole framework, based on the active component strategy of system pharmacology, is shown in Fig. 2.

## 3. Results

### 3.1 Potential target information for five components in XF

Eight components in XF were initially selected to uncover potential target proteins. Of these, five components, namely cleomiscosin A, myristic acid, succinic acid, xanthosine, and sitostenone, were successfully analyzed. A total of 99 target proteins were related to these five XF components as shown in Table 1. The frequency of some target proteins was much higher than that of others; high frequencies were obtained for P15086, P07360, P07195, MAOM_HUMAN (P23368), P35558, P35520, ACE_HUMAN (P12821), C1S_HUMAN (P09871), PH4H_HUMAN (P00439), FPPS_HUMAN (P14324), P50613, P12724, IMPA1_HUMAN (P29218), HXK1_HUMAN (P19367), P14061, and MCR_HUMAN (P08235).

**Table 1:** Potential targets of 5 effect components in XF

No.	Compound	Protein code	Protein name	Frequency
1	Cleomiscosin A	P06276	CHLE_HUMAN	3
2	Cleomiscosin A	P23141	EST1_HUMAN	3
3	Cleomiscosin A	P62937	P62937	3
4	Cleomiscosin A	P00918	CAH2_HUMAN	3
5	Cleomiscosin A	P24941	P24941	3
6	Cleomiscosin A	P07339	CATD_HUMAN	3
7	Cleomiscosin A	P03372	ESR1_HUMAN	3
8	Cleomiscosin A	Q15078	CD5R1_HUMAN	3
9	Cleomiscosin A	P00915	CAH1_HUMAN	3
10	Cleomiscosin A	P04062	GLCM_HUMAN	3
11	Cleomiscosin A	P11309	PIM1_HUMAN	3
12	Cleomiscosin A	P00491	PNPH_HUMAN	3
13	Cleomiscosin A	Q9NP99	Q9NP99	3
14	Cleomiscosin A	O14965	STK6_HUMAN	3
15	Cleomiscosin A	Q16539	Q16539	4
16	Cleomiscosin A	Q92731	ESR2_HUMAN	3
17	Cleomiscosin A	Q07343	PDE4B_HUMAN	4
18	Cleomiscosin A	O14757	CHK1_HUMAN	4
19	Cleomiscosin A	P45983	MK08_HUMAN	4
20	Cleomiscosin A	P08758	ANXA5_HUMAN	4
21	Myristic acid	P12643	BMP2_HUMAN	3
22	Myristic acid	P28482	MK01_HUMAN	3
23	Myristic acid	P09211	GSTP1_HUMAN	3
24	Myristic acid	P15121	ALDR_HUMAN	3
25	Myristic acid	P49137	P49137	3
26	Myristic acid	P10828	P10828	3
27	Myristic acid	P11309	P11309	3
28	Myristic acid	P27338	AOFB_HUMAN	3
29	Myristic acid	P62937	P62937	3
30	Myristic acid	P02774	VTDB_HUMAN	4
31	Myristic acid	P02768	ALBU_HUMAN	3
32	Myristic acid	P52732	KIF11_HUMAN	4
33	Myristic acid	P02652	APOA2_HUMAN	3
34	Myristic acid	P00918	CAH2_HUMAN	3
35	Myristic acid	P08842	STS_HUMAN	3
36	Myristic acid	P02766	TTHY_HUMAN	3
37	Myristic acid	Q14994	NR1I3_HUMAN	3
38	Myristic acid	P37231	PPARG_HUMAN	3
39	Myristic acid	P30044	PRDX5_HUMAN	3
40	Succinic acid	P09012	P09012	3
41	Succinic acid	P02743	P02743	4
42	Succinic acid	P12931	SRC_HUMAN	4
43	Succinic acid	O15382	O15382	4
44	Succinic acid	P18031	PTN1_HUMAN	4
45	Succinic acid	P15086	P15086	5
46	Succinic acid	P07360	P07360	5
47	Succinic acid	P02788	TRFL_HUMAN	4
48	Succinic acid	P03950	ANGI_HUMAN	4
49	Succinic acid	P07195	P07195	5
50	Succinic acid	P23368	MAOM_HUMAN	5
51	Succinic acid	Q9P2W7	B3GA1_HUMAN	4
52	Succinic acid	P35558	P35558	6
53	Succinic acid	P35520	P35520	7
54	Succinic acid	P12821	ACE_HUMAN	7
55	Succinic acid	P09871	C1S_HUMAN	6
56	Succinic acid	P00439	PH4H_HUMAN	5
57	Succinic acid	P14324	FPPS_HUMAN	8
58	Succinic acid	P50613	P50613	8
59	Succinic acid	P12724	P12724	6
60	Xanthosine	Q9BW91	Q9BW91	3
61	Xanthosine	P37173	TGFR2_HUMAN	3
62	Xanthosine	P04062	GLCM_HUMAN	3
63	Xanthosine	O14965	STK6_HUMAN	3
64	Xanthosine	Q13126	Q13126	3
65	Xanthosine	P00533	EGFR_HUMAN	3
66	Xanthosine	P24941	P24941	4
67	Xanthosine	Q07343	PDE4B_HUMAN	3
68	Xanthosine	P00915	CAH1_HUMAN	3
69	Xanthosine	Q12884	SEPR_HUMAN	3
70	Xanthosine	O14757	CHK1_HUMAN	3
71	Xanthosine	Q05315	LPPL_HUMAN	3
72	Xanthosine	P04745	P04745	3
73	Xanthosine	P18075	BMP7_HUMAN	4
74	Xanthosine	P03950	ANGI_HUMAN	4
75	Xanthosine	P00491	PNPH_HUMAN	4
76	Xanthosine	P29218	IMPA1_HUMAN	5
77	Xanthosine	Q99933	BAG1_HUMAN	4
78	Xanthosine	P19367	HXK1_HUMAN	5
79	Xanthosine	P17707	DCAM_HUMAN	4
80	Sitostenone	P52895	AK1C2_HUMAN	3
81	Sitostenone	P49137	P49137	3
82	Sitostenone	P55210	CASP7_HUMAN	3
83	Sitostenone	P12643	BMP2_HUMAN	3
84	Sitostenone	P08842	STS_HUMAN	3
85	Sitostenone	P27338	AOFB_HUMAN	3
86	Sitostenone	P02774	VTDB_HUMAN	4
87	Sitostenone	P11309	P11309	3
88	Sitostenone	P02768	ALBU_HUMAN	3
89	Sitostenone	P28482	MK01_HUMAN	3
90	Sitostenone	P45452	MMP13_HUMAN	4
91	Sitostenone	P10828	P10828	3
92	Sitostenone	P52732	KIF11_HUMAN	3
93	Sitostenone	P00918	CAH2_HUMAN	3
94	Sitostenone	P14061	P14061	5
95	Sitostenone	P02652	APOA2_HUMAN	3
96	Sitostenone	P08235	MCR_HUMAN	5
97	Sitostenone	P06401	PRGR_HUMAN	4
98	Sitostenone	P10275	ANDR_HUMAN	3
99	Sitostenone	P02766	TTHY_HUMAN	3

### 3.2 Pathway analysis of potential target proteins

The potential pathway information for the five effect components in XF is shown in Table 2.

**Table 2:** The potential pathways targeted by 5 effect components in XF

No. of pathway	Pathway name	Frequency
Pw1	Nuclear receptor transcription pathway	1
Pw2	Activation of the AP-1 family of transcription factors	3
Pw3	MAPK targets/nuclear events mediated by MAP kinases	10
Pw4	p38MAPK events	3
Pw5	Generic Transcription Pathway	42
Pw6	Transcriptional regulation by RUNX2	10
Pw7	Signalling to RAS	3
Pw8	RNA polymerase II transcription	73
Pw9	Regulation of TP53 Activity through phosphorylation	5
Pw10	Metabolism	68
Pw11	Nuclear events (kinase and transcription factor activation)	7
Pw12	RUNX2 regulates osteoblast differentiation	5
Pw13	Metabolism of steroids	23
Pw14	MAP kinase activation in TLR cascade	15
Pw15	Erythrocytes take up oxygen and release carbon dioxide	1
Pw16	RUNX2 regulates bone development	5
Pw17	Signalling to ERKs	3
Pw18	Gene expression (transcription)	80
Pw19	Interleukin-17 signaling	15
Pw20	Digestion of dietary carbohydrate	2
Pw21	Gene and protein expression by JAK-STAT signaling after Interleukin-12 stimulation	3
Pw22	DSCAM interactions	2
Pw23	NGF signalling *via* TRKA from the plasma membrane	10
Pw24	Reversible hydration of carbon dioxide	2
Pw25	O_2_/CO_2_ exchange in erythrocytes	2
Pw26	Erythrocytes take up carbon dioxide and release oxygen	1
Pw27	Cellular responses to stress	27
Pw28	MyD88 cascade initiated on plasma membrane	15
Pw29	Toll like receptor 10 (TLR10) cascade	15
Pw30	Toll like receptor 5 (TLR5) cascade	15
Pw31	TRAF6 mediated induction of NFkB and MAP kinases upon TLR7/8 or 9 activation	15
Pw32	Platelet activation, signaling and aggregation	32
Pw33	Oxidative stress induced senescence	12
Pw34	MyD88 dependent cascade initiated on endosome	15
Pw35	Toll like receptor 7/8 (TLR7/8) cascade	15
Pw36	MyD88:Mal cascade initiated on plasma membrane	15
Pw37	Toll like receptor TLR6:TLR2 cascade	15
Pw38	Spry regulation of FGF signaling	2
Pw39	Netrin-1 signaling	12
Pw40	Toll like receptor 9 (TLR9) cascade	15
Pw41	Toll like receptor 3 (TLR3) cascade	15
Pw42	Toll like receptor TLR1:TLR2 cascade	15
Pw43	Toll like receptor 2 (TLR2) cascade	15
Pw44	TRIF(TICAM1)-mediated TLR4 signaling	15
Pw45	MyD88-independent TLR4 cascade	15
Pw46	Defective HK1 causes hexokinase deficiency (HK deficiency)	1
Pw47	Metabolism of angiotensinogen to angiotensins	4
Pw48	Regulation of TP53 Activity	6
Pw49	Signaling by receptor tyrosine kinases	81
Pw50	Cellular senescence	21
Pw51	HSP90 chaperone cycle for steroid hormone receptors (SHR)	3
Pw52	Interleukin-12 family signaling	3

### 3.3 Main effect component-target protein-pathway network construction for XF

**Fig. 3:**
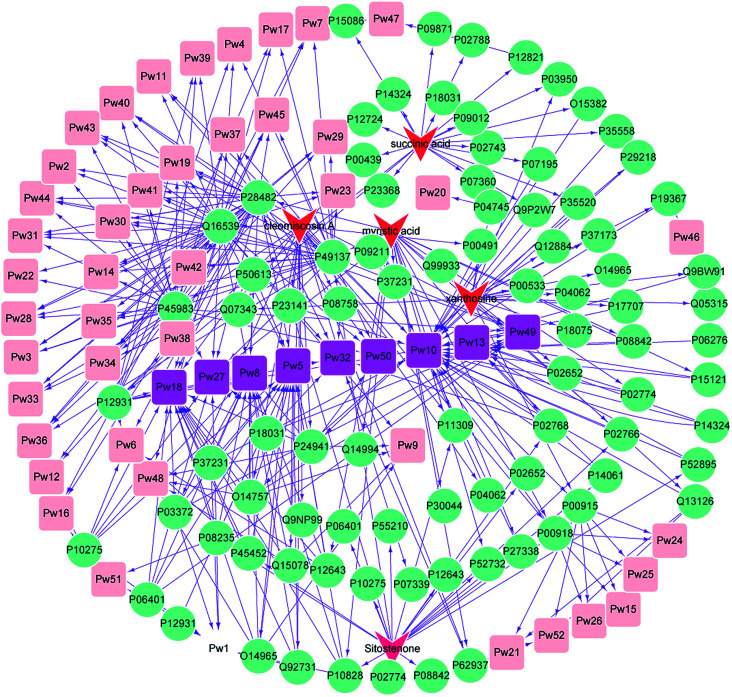
Component-target-pathway network of XF.

An effect component-target-pathway network model was established using Cytoscape software, and the relationship between the 5 components, 99 targets and 52 pathways is shown in Fig. 3. There were complex network relationships between the effect components of XF and their targets, as well as the targets and pathways.

Cleomiscosin A was related to the following pathways: nuclear receptor transcription pathway (Pw1), activation of the AP-1 family of transcription factors (Pw2), MAPK targets/nuclear events mediated by MAP kinases (Pw3), p38MAPK events (Pw4), Generic Transcription Pathway (Pw5), Transcriptional regulation by RUNX2 (Pw6), Signalling to RAS (Pw7), RNA Polymerase II Transcription (Pw8), Regulation of TP53 Activity through Phosphorylation (Pw9), Metabolism (Pw10), Nuclear Events (kinase and transcription factor activation) (Pw11), MAP kinase activation in TLR cascade (Pw14), erythrocytes take up oxygen and release carbon dioxide (Pw15), Signalling to ERKs (Pw17), Gene expression (Transcription) (Pw18), Interleukin-17 signaling (Pw19), Gene and protein expression by JAK-STAT signaling after Interleukin-12 stimulation (Pw21), DSCAM interactions (Pw22), NGF signalling *via* TRKA from the plasma membrane (Pw23), Reversible hydration of carbon dioxide (Pw24), O_2_/CO_2_ exchange in erythrocytes (Pw25), erythrocytes take up carbon dioxide and release oxygen (Pw26), cellular responses to stress (Pw27), MyD88 cascade initiated on plasma membrane (Pw28), Toll Like Receptor 10 (TLR10) Cascade (Pw29), Toll Like Receptor 5 (TLR5) Cascade (Pw30), TRAF6 mediated induction of NFkB and MAP kinases upon TLR7/8 or 9 activation (Pw31), platelet activation, signaling and aggregation (Pw32), oxidative stress induced senescence (Pw33), MyD88 dependent cascade initiated on endosome (Pw34), Toll Like Receptor 7/8 (TLR7/8) Cascade (Pw35), MyD88:Mal cascade initiated on plasma membrane (Pw36), Toll Like Receptor TLR6:TLR2 Cascade (Pw37), Netrin-1 signaling (Pw39), Toll Like Receptor 9 (TLR9) Cascade (Pw40), Toll Like Receptor 3 (TLR3) Cascade (Pw41), Toll Like Receptor TLR1:TLR2 Cascade (Pw42), Toll Like Receptor 2 (TLR2) Cascade (Pw43), TRIF(TICAM1)-mediated TLR4 signaling (Pw44), MyD88-independent TLR4 cascade (Pw45), Regulation of TP53 Activity (Pw48), Signaling by Receptor Tyrosine Kinases (Pw49), Cellular Senescence (Pw50) and Interleukin-12 family signaling (Pw52).

Myristic acid was related to the following pathways: nuclear receptor transcription pathway (Pw1), Activation of the AP-1 family of transcription factors (Pw2), MAPK targets/nuclear events mediated by MAP kinases (Pw3), p38MAPK events (Pw4), Generic Transcription Pathway (Pw5), Transcriptional regulation by RUNX2 (Pw6), Signalling to RAS (Pw7), RNA Polymerase II Transcription (Pw8), Metabolism (Pw10), Nuclear Events (kinase and transcription factor activation) (Pw11), RUNX2 regulates osteoblast differentiation (Pw12), Metabolism of steroids (Pw13), MAP kinase activation in TLR cascade (Pw14), RUNX2 regulates bone development (Pw16), Signalling to ERKs (Pw17), Gene expression (Transcription) (Pw18), Interleukin-17 signaling (Pw19), NGF signalling *via* TRKA from the plasma membrane (Pw23), Cellular responses to stress (Pw27), MyD88 cascade initiated on plasma membrane (Pw28), Toll Like Receptor 10 (TLR10) Cascade (Pw29), Toll Like Receptor 5 (TLR5) Cascade (Pw30), TRAF6 mediated induction of NFkB and MAP kinases upon TLR7/8 or 9 activation (Pw31), Platelet activation, signaling and aggregation (Pw32), Oxidative Stress Induced Senescence (Pw33), MyD88 dependent cascade initiated on endosome (Pw34), Toll Like Receptor 7/8 (TLR7/8) Cascade (Pw35), MyD88:Mal cascade initiated on plasma membrane (Pw36), Toll Like Receptor TLR6:TLR2 Cascade (Pw37), Spry regulation of FGF signaling (Pw38), Toll Like Receptor 9 (TLR9) Cascade (Pw40), Toll Like Receptor 3 (TLR3) Cascade (Pw41), Toll Like Receptor TLR1:TLR2 Cascade (Pw42), Toll Like Receptor 2 (TLR2) Cascade (Pw43), TRIF(TICAM1)-mediated TLR4 signaling (Pw44), MyD88-independent TLR4 cascade (Pw45), Signaling by Receptor Tyrosine Kinases (Pw49) and Cellular Senescence (Pw50).

Succinic acid was related to the following pathways: p38MAPK events (Pw4), Generic Transcription Pathway (Pw5), Transcriptional regulation by RUNX2 (Pw6), Signalling to RAS (Pw7), RNA Polymerase II Transcription (Pw8), Metabolism (Pw10), RUNX2 regulates osteoblast differentiation (Pw12), Metabolism of steroids (Pw13), RUNX2 regulates bone development (Pw16), Signalling to ERKs (Pw17), Gene expression (Transcription) (Pw18), NGF signalling *via* TRKA from the plasma membrane (Pw23), Platelet activation, signaling and aggregation (Pw32), Spry regulation of FGF signaling (Pw38), Netrin-1 signaling (Pw39), Metabolism of Angiotensinogen to Angiotensins (Pw47) and Signaling by Receptor Tyrosine Kinases (Pw49).

Xanthosine was related to the following pathways: Metabolism (Pw10), Gene and protein expression by JAK-STAT signaling after Interleukin-12 stimulation (Pw21), Defective HK1 causes hexokinase deficiency (HK deficiency) (Pw46) and Interleukin-12 family signaling (Pw52).

Sitostenone was related to the following pathways: nuclear receptor transcription pathway (Pw1), Generic Transcription Pathway (Pw5), transcriptional regulation by RUNX2 (Pw6), Signalling to RAS (Pw7), RNA Polymerase II Transcription (Pw8), Metabolism (Pw10), Nuclear Events (kinase and transcription factor activation) (Pw11), RUNX2 regulates osteoblast differentiation (Pw12), Metabolism of steroids (Pw13), RUNX2 regulates bone development (Pw16), Gene expression (Transcription) (Pw18), Interleukin-17 signaling (Pw19), Cellular responses to stress (Pw27), Signaling by Receptor Tyrosine Kinases (Pw49) and HSP90 chaperone cycle for steroid hormone receptors (SHR) (Pw51).

We were surprised to find that the five components have one common pathway – the Metabolism pathway (Pw10). Nine other pathways occurred frequently including Generic Transcription Pathway (Pw5), RNA Polymerase II Transcription (Pw8), Metabolism (Pw10), Metabolism of steroids (Pw13), Gene expression (Transcription) (Pw18), Cellular responses to stress (Pw27), Platelet activation, signaling and aggregation (Pw32), Signaling by Receptor Tyrosine Kinases (Pw49) and Cellular Senescence (Pw50).

## 4. Discussion

The PharmMapper database can be used to search for potential targets based on small active molecules. This database uses a pharmacophore matching method to obtain drug point information by rapidly searching four major databases. This database is based on 7000 pharmacophore models and can cover most clinical indications.

According to the network pharmacological prediction of the five components in XF, all five components can be connected with the same pathway *via* the same target, and also can be connected with the same pathways with different targets. Different components can produce the same effect through different ways, and also can offer multi-target synergy.

Interestingly, this predicted common pathway is consistent with the result we got from the metabolic pathway analysis experiment (unpublished), which indicates that this result is reliable although it still requires further verification.

## 5. Conclusion

In this paper, a network pharmacology method has been successfully developed to predict the potential targets of the main components absorbed in serum after oral administration of XF extract. When considered alongside our previous anti-allergic rhinitis metabolomics study, the predicted potential targets and the role of the pathways were considered to have a certain degree of accuracy. This article has established a “multi component-multi target-multi pathway” network model for TCM research, and started to unravel the multidimensional regulatory action of XF, which may provide a reference and basis for studying the molecular mechanism of XF.

## Conflicts of interest

The authors have declared no conflicts of interest.

## Supplementary Material

RA-008-C8RA00186C-s001

## References

[cit1] Wang J., Li X. J. (2010). Drug targets discovery based on dynamic signal transduction networks. Acta Pharm. Sin..

[cit2] Wang N., Zheng Y., Gu J., Cai Y., Wang S., Zhang F., Chen J., Situ H., Lin Y., Wang Z. (2017). Network-pharmacology-based validation of TAMS/CXCL-1 as key mediator of XIAOPI formula preventing breast cancer development and metastasis. Sci. Rep..

[cit3] Lyu M., Yan C. L., Liu H. X., Wang T. Y., Shi X. H., Liu J. P., Orgah J., Fan G. W., Han J. H., Wang X. Y., Zhu Y. (2017). Network pharmacology exploration reveals endothelial inflammation as a common mechanism for stroke and coronary artery disease treatment of Danhong injection. Sci. Rep..

[cit4] Hong M., Li S., Tan H. Y., Cheung F., Wang N., Huang J., Feng Y. (2017). A Network-Based Pharmacology Study of the Herb-Induced Liver Injury Potential of Traditional Hepatoprotective Chinese Herbal Medicines. Molecules.

[cit5] Yue S. J., Xin L. T., Fan Y. C., Li S. J., Tang Y. P., Duan J. A., Guan H. S., Wang C. Y. (2017). Herb pair Danggui-Honghua: mechanisms underlying blood stasis syndrome by system pharmacology approach. Sci. Rep..

[cit6] Hong M., Zhang Y. S., Li S., Tan H. Y., Wang N., Mu S., Hao X., Feng Y. (2017). A Network Pharmacology-Based Study on the Hepatoprotective Effect of Fructus Schisandrae. Molecules.

[cit7] Zhang A. H., Fang H., Wang Y. Y., Yan G. L., Sun H., Zhou X. H., Wang Y. Y., Liu L., Wang X. J. (2017). Discovery and verification of the potential targets from bioactive molecules by network pharmacology-based target prediction combined with high-throughput metabolomics. RSC Adv..

[cit8] Kang G. L., Li S., Zhang J. F. (2008). Entropy-based model for interpreting life systems in traditional Chinese medicine. Evid. Based Complement. Alternat. Med..

[cit9] Wu M., Ma C. H., Wu Y., Li S. (2008). Simultaneous LC analysis of five bioactive alkaloids in an anti-angiogenesis herbal formula. Chromatographia.

[cit10] Li S. (2009). Network systems underlying traditional Chinese medicine syndrome and herb formula. Curr. Bioinf..

[cit11] Li S., Zhang B., Zhang N. B. (2011). Network target for screening synergistic drug combinations with application to traditional Chinese medicine. BMC Syst. Biol..

[cit12] Chinese Pharmacopoeia Commission Pharmacopoeia of the People's Republic of China, China Medical Science and Technology Press, Beijing, 2015, p. 162, Part I

[cit13] Cui X. R., Ma X. B., Zhang Q., Li Q. S., Wang W., Han Q. J., Lei H. M., Li Q. (2012). Research progress on the chemical composition and clinical application of Xanthii Fructus. Drugs Clin..

