# Supplementary material for: Prediction of the targets of the main components in blood after oral administration of Xanthii Fructus: a network pharmacology study
Source: RSC Adv. 2018 Feb 27;8(16):8870–7. doi: 10.1039/c8ra00186c (PMC9078587; doi:10.1039/c8ra00186c)
Supplement: RA-008-C8RA00186C-s001 [file RA-008-C8RA00186C-s001.pdf]

Electronic Supplementary Material (ESI) for RSC Advances.
This journal is © The Royal Society of Chemistry 2018

The raw data was shown in the following table 1-3. The potential protein got from PharmMapper database was shown in table1. Then the potential protein was imported to Reactome database and the protein pathway was collected, the result was shown in table2. The component, protein and pathway were collected together before imported into the cytoscape software, the collected data was shown in table3.

Table1 Data got from PharmMapper database

component	protein
cleomiscosin A	P06276
cleomiscosin A	P23141
cleomiscosin A	P62937
cleomiscosin A	P00918
cleomiscosin A	P24941
cleomiscosin A	P07339
cleomiscosin A	P03372
cleomiscosin A	Q15078
cleomiscosin A	P00915
cleomiscosin A	P04062
cleomiscosin A	P11309
cleomiscosin A	P00491
cleomiscosin A	Q9NP99
cleomiscosin A	O14965
cleomiscosin A	Q16539
cleomiscosin A	Q92731
cleomiscosin A	Q07343
cleomiscosin A	O14757
cleomiscosin A	P45983
cleomiscosin A	P08758
myristic acid	P12643
myristic acid	P28482
myristic acid	P09211
myristic acid	P15121
myristic acid	P49137
myristic acid	P10828
myristic acid	P11309
myristic acid	P27338
myristic acid	P62937
myristic acid	P02774
myristic acid	P02768
myristic acid	P52732
myristic acid	P02652
myristic acid	P00918
myristic acid	P08842
myristic acid	P02766

myristic acid	Q14994
myristic acid	P37231
myristic acid	P30044
succinic acid	P09012
succinic acid	P02743
succinic acid	P12931
succinic acid	O15382
succinic acid	P18031
succinic acid	P15086
succinic acid	P07360
succinic acid	P02788
succinic acid	P03950
succinic acid	P07195
succinic acid	P23368
succinic acid	Q9P2W7
succinic acid	P35558
succinic acid	P35520
succinic acid	P12821
succinic acid	P09871
succinic acid	P00439
succinic acid	P14324
succinic acid	P50613
succinic acid	P12724
xanthosine	Q9BW91
xanthosine	P37173
xanthosine	P04062
xanthosine	O14965
xanthosine	Q13126
xanthosine	P00533
xanthosine	P24941
xanthosine	Q07343
xanthosine	P00915
xanthosine	Q12884
xanthosine	O14757
xanthosine	Q05315
xanthosine	P04745
xanthosine	P18075
xanthosine	P03950
xanthosine	P00491
xanthosine	P29218
xanthosine	Q99933
xanthosine	P19367
xanthosine	P17707
Sitostenone	P52895

Sitostenone	P49137
Sitostenone	P55210
Sitostenone	P12643
Sitostenone	P08842
Sitostenone	P27338
Sitostenone	P02774
Sitostenone	P11309
Sitostenone	P02768
Sitostenone	P28482
Sitostenone	P45452
Sitostenone	P10828
Sitostenone	P52732
Sitostenone	P00918
Sitostenone	P14061
Sitostenone	P02652
Sitostenone	P08235
Sitostenone	P06401
Sitostenone	P10275
Sitostenone	P02766

Table2 Data got from Reactome database

protein	pathway
P37231;P10828;Q92731;P03372;P10275;P06401;P08235	Nuclear Receptor transcription pathway
Q16539;P28482;P45983	Activation of the AP-1 family of transcription factors
Q16539;P28482;P45983;P49137	MAPK targets/ Nuclear events mediated by MAP kinases
Q16539;P12931;P49137	p38MAPK events
Q16539;P10828;P28482;P50613;P12643;P12931;P10275;P37231;Q92731;P24941;P45452;P03372;P06401;P08235;O14757;O14965;Q15078	Generic Transcription Pathway
P28482;P12643;P45452;P03372;P12931;P10275	Transcriptional regulation by RUNX2
Q16539;P12931;P49137	Signalling to RAS
Q16539;P10828;P28482;P50613;P12643;P12931;P10275;P37231;Q92731;P24941;P45452;P03372;P06401;P08235;O14757;O14965;Q15078	RNA Polymerase II Transcription
Q16539;P24941;O14757;Q15078;O14965	Regulation of TP53 Activity through Phosphorylation

O15382;P52895;P00491;P35558;Q9BW91;P14061;P07195;P04062;P02768;P17707;P02766;P49137;P09211;P08842;Q13126;P23141;P35520;Q9P2W7;P14324;P19367;P00918;P37231;P00915;P15121;P06276;P29218;P02774;P27338;P02652	Metabolism
Q16539;P28482;P49137	Nuclear Events (kinase and transcription factor activation)
P28482;P12931;P10275	RUNX2 regulates osteoblast differentiation
P52895;P15121;P14061;P02768;P14324;P02774	Metabolism of steroids
Q16539;P28482;P45983;P49137	MAP kinase activation in TLR cascade
P00915;P00918	Erythrocytes take up oxygen and release carbon dioxide
P28482;P12931;P10275	RUNX2 regulates bone development
Q16539;P12931;P49137	Signalling to ERKs
Q16539;P10828;P28482;P50613;P12643;P12931;P10275;P37231;Q92731;P24941;P45452;P03372;P06401;P08235;O14757;O14965;Q15078	Gene expression (Transcription)
Q16539;P28482;P45983;P49137	Interleukin-17 signaling
P04745	Digestion of dietary carbohydrate
Q13126;P00915;P62937	Gene and protein expression by JAK-STAT signaling after Interleukin-12 stimulation
Q16539;P45983	DSCAM interactions
Q16539;P28482;P12931;P49137	NGF signalling via TRKA from the plasma membrane
P00915;P00918	Reversible hydration of carbon dioxide
P00915;P00918	O2/CO2 exchange in erythrocytes
P00915;P00918	Erythrocytes take up carbon dioxide and release oxygen
Q16539;P28482;P24941;P45983;P49137;P09211;P10275;P06401;P08235	Cellular responses to stress
Q16539;P28482;P45983;P49137	MyD88 cascade initiated on plasma membrane
Q16539;P28482;P45983;P49137	Toll Like Receptor 10 (TLR10) Cascade
Q16539;P28482;P45983;P49137	Toll Like Receptor 5 (TLR5) Cascade
Q16539;P28482;P45983;P49137	TRAF6 mediated induction of NFkB and MAP kinases upon TLR7/8 or 9 activation
P08758;Q16539;P62937;P28482;P18031;P02768;P12931	Platelet activation, signaling and aggregation
Q16539;P28482;P45983;P49137	Oxidative Stress Induced Senescence
Q16539;P28482;P45983;P49137	MyD88 dependent cascade initiated on

	endosome
Q16539;P28482;P45983;P49137	Toll Like Receptor 7/8 (TLR7/8) Cascade
Q16539;P28482;P45983;P49137	MyD88:Mal cascade initiated on plasma membrane
Q16539;P28482;P45983;P49137	Toll Like Receptor TLR6:TLR2 Cascade
P28482;P12931	Spry regulation of FGF signaling
Q16539;P45983;P12931	Netrin-1 signaling
Q16539;P28482;P45983;P49137	Toll Like Receptor 9 (TLR9) Cascade
Q16539;P28482;P45983;P49137	Toll Like Receptor 3 (TLR3) Cascade
Q16539;P28482;P45983;P49137	Toll Like Receptor TLR1:TLR2 Cascade
Q16539;P28482;P45983;P49137	Toll Like Receptor 2 (TLR2) Cascade
Q16539;P28482;P45983;P49137	TRIF(TICAM1)-mediated TLR4 signaling
Q16539;P28482;P45983;P49137	MyD88-independent TLR4 cascade
P19367	Defective HK1 causes hexokinase deficiency (HK deficiency)
P15086;P12821	Metabolism of Angiotensinogen to Angiotensins
Q16539;P24941;O14757;Q15078;O14965	Regulation of TP53 Activity
Q16539;P28482;P18031;P45983;P03372;P49137;P12931;O14757;P06401	Signaling by Receptor Tyrosine Kinases
Q16539;P28482;P24941;P45983;P49137	Cellular Senescence
P10275;P08235;P06401	HSP90 chaperone cycle for steroid hormone receptors (SHR)
Q13126;P00915;P62937	Interleukin-12 family signaling

Table 3 Data imported to cytoscape

componet-protein	protein-pathway
cleomiscosin A	P06276
cleomiscosin A	P23141
cleomiscosin A	P62937
cleomiscosin A	P00918
cleomiscosin A	P24941
cleomiscosin A	P07339
cleomiscosin A	P03372
cleomiscosin A	Q15078
cleomiscosin A	P00915
cleomiscosin A	P04062
cleomiscosin A	P11309
cleomiscosin A	P00491
cleomiscosin A	Q9NP99
cleomiscosin A	O14965
cleomiscosin A	Q16539
cleomiscosin A	Q92731
cleomiscosin A	Q07343
cleomiscosin A	O14757
cleomiscosin A	P45983
cleomiscosin A	P08758
myristic acid	P12643
myristic acid	P28482
myristic acid	P09211
myristic acid	P15121
myristic acid	P49137
myristic acid	P10828
myristic acid	P11309
myristic acid	P27338
myristic acid	P62937
myristic acid	P02774
myristic acid	P02768
myristic acid	P52732
myristic acid	P02652
myristic acid	P00918
myristic acid	P08842
myristic acid	P02766
myristic acid	Q14994
myristic acid	P37231
myristic acid	P30044
succinic acid	P09012
succinic acid	P02743

succinic acid	P12931
succinic acid	O15382
succinic acid	P18031
succinic acid	P15086
succinic acid	P07360
succinic acid	P02788
succinic acid	P03950
succinic acid	P07195
succinic acid	P23368
succinic acid	Q9P2W7
succinic acid	P35558
succinic acid	P35520
succinic acid	P12821
succinic acid	P09871
succinic acid	P00439
succinic acid	P14324
succinic acid	P50613
succinic acid	P12724
xanthosine	Q9BW91
xanthosine	P37173
xanthosine	P04062
xanthosine	O14965
xanthosine	Q13126
xanthosine	P00533
xanthosine	P24941
xanthosine	Q07343
xanthosine	P00915
xanthosine	Q12884
xanthosine	O14757
xanthosine	Q05315
xanthosine	P04745
xanthosine	P18075
xanthosine	P03950
xanthosine	P00491
xanthosine	P29218
xanthosine	Q99933
xanthosine	P19367
xanthosine	P17707
Sitostenone	P52895
Sitostenone	P49137
Sitostenone	P55210
Sitostenone	P12643
Sitostenone	P08842
Sitostenone	P27338

Sitostenone	P02774
Sitostenone	P11309
Sitostenone	P02768
Sitostenone	P28482
Sitostenone	P45452
Sitostenone	P10828
Sitostenone	P52732
Sitostenone	P00918
Sitostenone	P14061
Sitostenone	P02652
Sitostenone	P08235
Sitostenone	P06401
Sitostenone	P10275
Sitostenone	P02766
P37231	Pw1
P10828	Pw1
Q92731	Pw1
P03372	Pw1
P10275	Pw1
P06401	Pw1
P08235	Pw1
Q16539	Pw2
P28482	Pw2
P45983	Pw2
Q16539	Pw3
P28482	Pw3
P45983	Pw3
P49137	Pw3
Q16539	Pw4
P12931	Pw4
P49137	Pw4
Q16539	Pw5
P10828	Pw5
P28482	Pw5
P50613	Pw5
P12643	Pw5
P12931	Pw5
P10275	Pw5
P37231	Pw5
Q92731	Pw5
P24941	Pw5
P45452	Pw5
P03372	Pw5
P06401	Pw5

P08235	Pw5
O14757	Pw5
O14965	Pw5
Q15078	Pw5
P28482	Pw6
P12643	Pw6
P45452	Pw6
P03372	Pw6
P12931	Pw6
P10275	Pw6
Q16539	Pw7
P12931	Pw7
P49137	Pw7
Q16539	Pw8
P10828	Pw8
P28482	Pw8
P50613	Pw8
P12643	Pw8
P12931	Pw8
P10275	Pw8
P37231	Pw8
Q92731	Pw8
P24941	Pw8
P45452	Pw8
P03372	Pw8
P06401	Pw8
P08235	Pw8
O14757	Pw8
O14965	Pw8
Q15078	Pw8
Q16539	Pw9
P24941	Pw9
O14757	Pw9
Q15078	Pw9
O14965	Pw9
O15382	Pw10
P52895	Pw10
P00491	Pw10
P35558	Pw10
Q9BW91	Pw10
P14061	Pw10
P07195	Pw10
P04062	Pw10
P02768	Pw10

P17707	Pw10
P02766	Pw10
P49137	Pw10
P09211	Pw10
P08842	Pw10
Q13126	Pw10
P23141	Pw10
P35520	Pw10
Q9P2W7	Pw10
P14324	Pw10
P19367	Pw10
P00918	Pw10
P37231	Pw10
P00915	Pw10
P15121	Pw10
P06276	Pw10
P29218	Pw10
P02774	Pw10
P27338	Pw10
P02652	Pw10
Q16539	Pw11
P28482	Pw11
P49137	Pw11
P28482	Pw12
P12931	Pw12
P10275	Pw12
P52895	Pw13
P15121	Pw13
P14061	Pw13
P02768	Pw13
P14324	Pw13
P02774	Pw13
Q16539	Pw14
P28482	Pw14
P45983	Pw14
P49137	Pw14
P00915	Pw15
P00918	Pw15
P28482	Pw16
P12931	Pw16
P10275	Pw16
Q16539	Pw17
P12931	Pw17
P49137	Pw17

Q16539	Pw18
P10828	Pw18
P28482	Pw18
P50613	Pw18
P12643	Pw18
P12931	Pw18
P10275	Pw18
P37231	Pw18
Q92731	Pw18
P24941	Pw18
P45452	Pw18
P03372	Pw18
P06401	Pw18
P08235	Pw18
O14757	Pw18
O14965	Pw18
Q15078	Pw18
Q16539	Pw19
P28482	Pw19
P45983	Pw19
P49137	Pw19
P04745	Pw20
Q13126	Pw21
P00915	Pw21
P62937	Pw21
Q16539	Pw22
P45983	Pw22
Q16539	Pw23
P28482	Pw23
P12931	Pw23
P49137	Pw23
P00915	Pw24
P00918	Pw24
P00915	Pw25
P00918	Pw25
P00915	Pw26
P00918	Pw26
Q16539	Pw27
P28482	Pw27
P24941	Pw27
P45983	Pw27
P49137	Pw27
P09211	Pw27
P10275	Pw27

P06401	Pw27
P08235	Pw27
Q16539	Pw28
P28482	Pw28
P45983	Pw28
P49137	Pw28
Q16539	Pw29
P28482	Pw29
P45983	Pw29
P49137	Pw29
Q16539	Pw30
P28482	Pw30
P45983	Pw30
P49137	Pw30
Q16539	Pw31
P28482	Pw31
P45983	Pw31
P49137	Pw31
P08758	Pw32
Q16539	Pw32
P62937	Pw32
P28482	Pw32
P18031	Pw32
P02768	Pw32
P12931	Pw32
Q16539	Pw33
P28482	Pw33
P45983	Pw33
P49137	Pw33
Q16539	Pw34
P28482	Pw34
P45983	Pw34
P49137	Pw34
Q16539	Pw35
P28482	Pw35
P45983	Pw35
P49137	Pw35
Q16539	Pw36
P28482	Pw36
P45983	Pw36
P49137	Pw36
Q16539	Pw37
P28482	Pw37
P45983	Pw37

P49137	Pw37
P28482	Pw38
P12931	Pw38
Q16539	Pw39
P45983	Pw39
P12931	Pw39
Q16539	Pw40
P28482	Pw40
P45983	Pw40
P49137	Pw40
Q16539	Pw41
P28482	Pw41
P45983	Pw41
P49137	Pw41
Q16539	Pw42
P28482	Pw42
P45983	Pw42
P49137	Pw42
Q16539	Pw43
P28482	Pw43
P45983	Pw43
P49137	Pw43
Q16539	Pw44
P28482	Pw44
P45983	Pw44
P49137	Pw44
Q16539	Pw45
P28482	Pw45
P45983	Pw45
P49137	Pw45
P19367	Pw46
P15086	Pw47
P12821	Pw47
Q16539	Pw48
P24941	Pw48
O14757	Pw48
Q15078	Pw48
O14965	Pw48
Q16539	Pw49
P28482	Pw49
P18031	Pw49
P45983	Pw49
P03372	Pw49
P49137	Pw49

P12931	Pw49
O14757	Pw49
P06401	Pw49
Q16539	Pw50
P28482	Pw50
P24941	Pw50
P45983	Pw50
P49137	Pw50
P10275	Pw51
P08235	Pw51
P06401	Pw51
Q13126	Pw52
P00915	Pw52
P62937	Pw52
